# Is Preventative Long-Segment Surgery for Multi-Level Spondylolysis Necessary? A Finite Element Analysis Study

**DOI:** 10.1371/journal.pone.0149707

**Published:** 2016-02-26

**Authors:** Jianqiang Mo, Wen Zhang, Dongyan Zhong, Hao Xu, Lan Wang, Jia Yu, Zongping Luo

**Affiliations:** Department of Orthopedics, The First Affiliated Hospital of Soochow University, Orthopedic Institute, Medical College, Soochow University, Suzhou, Jiangsu, People’s Republic of China; Mayo Clinic Minnesota, UNITED STATES

## Abstract

**Objective:**

For multi-level spondylolysis patients, surgeons commonly choose to fix all the segments with pars interarticularis defect even those without slippage and not responsible for clinical symptoms. In this study, we tried to study the necessity of the preventative long-segment surgery for the defected segment without slippage in treatment of multi-level spondylolysis patients from a biomechanical perspective.

**Method:**

We established a bi-level spondylolysis model with pars defects at L4 and L5 segments, and simulated posterior lumbar interbody fusion (PLIF) and pedicle screw fixation at L5-S1 level. Then we compared the biomechanical changes at L4 segment before and after surgery in neutral, flexion, extension, lateral bending and axial rotation position.

**Results:**

The stress on L4 pars interarticularis was very similar before and after surgery, and reached the highest in axial rotation. The L3-L4 intradiscal pressure was almost the same, while L4-L5 intradiscal pressure changed a little in lateral bending (increase from 1.993 to 2.160 MPa) and axial rotation (decrease from 1.639 to 1.307 MPa) after surgery. The PLIF surgery caused a little increase of range of motion at adjacent L4-L5 and L3-L4 levels, but the change is very tiny (1 degree).

**Conclusion:**

The PLIF surgery will not cause significant biomechanical change at adjacent segment with pars defect in multi-level spondylolysis. On the contrary, excessive long-segment surgery will damage surrounding soft tissues which are important for maintaining the stability of spine. So a preventative long-segment surgery is not necessary for multi-level spondylolysis as long as there are no soft tissue degeneration signs at adjacent level.

## Introduction

Spondylolysis is defined as a defect in the pars interarticularis (pars for short) of the vertebral arch. The morbidity of spondylolysis is about 3–6% in general population[1], and even higher in athletes[2]. The most vulnerable segments are L5-S1(85–95%) and L4-L5 (5–15%)[1]. When the defected vertebra develops to a forward displacement over its inferior vertebra, it’s called spondylolisthesis. Spondylolisthesis is a common cause of low back pain, and surgery is the main treatment for spondylolisthesis with severe symptoms.

Multi-level spondylolysis is comparatively rarer. Ravichandran[3] reported an incidence of about 1.5% amongst symptomatic patients. Sakai et al.[4] reported an incidence of 0.03% in general Japanese population. However, a couple of papers introduced their experience of treating multi-level spondylolysis patients[5–12]. Notably, Song et al.[12] reported the surgical treatment of 54 multi-level spondylolisthesis patients in 8 years. So it is still an issue worth paying attention to.

Some of the multi-level spondylolysis patients may only have one segment develops to spondylolisthesis and causes symptoms (responsible segment), the adjacent pars defects are just found casually by preoperative examination and were not responsible for the symptoms (innocent segment). Surgeons used to fix all the defected segments at the same time[12, 13]. It is reasonable to fix the responsible segment, but is a preventative fixation for the innocent segment without slippage necessary? There have been no biomechanical and clinical reports supporting previous surgeons’ choice.

By reviewing previous literatures, we found that the probability for spondylolysis to develop to spondylolisthesis which needs to be treated by surgery is quite low[14, 15]. So we assume that as long as the fixation of the responsible segment does not apply extra stress on the adjacent innocent segment, it can maintain its stability and does not need a preventative fixation.

It is difficult to set up a randomized controlled clinical trial due to the low incidence of multi-level spondylolysis. So we used finite element analysis (FEA) in this study to investigate the biomechanical influence of short-segment posterior lumbar interbody fusion (PLIF) on adjacent innocent segment in bi-level spondylolysis.

## Methods

A three dimensional (3D) FEA model of L3-S1 segments which had been established in our previous study was used, the validation of this model had been documented before[16]. The model was modified to simulate two situations. Before surgery: bilateral pars defects were simulated at L4 and L5 segments to establish a bi-level spondylolysis model; Short-segment PLIF: short-segment surgery (PLIF coupled with bilateral pedicle screw fixation) was performed at L5-S1 level on the bi-level spondylolysis model. By comparing these two models, we could see whether there would be biomechanical changes at the defected L4 segment after fixation of L5-S1 level in bi-level spondylolysis.

### Finite Element Model

The Computed Tomography scan images of a normal male adult’s lumbar spine (slice width 0.625 mm) were imported into Mimics 10.01 (Materialise NV, Belgium). A 3D model of L3-S1 vertebras and intervertebral discs was established after processes including thresholding, region growing, 3D modeling, and so on. The vertebras consisted of anterior centrum (cancellous and cortical bone) and posterior vertebral arch. The intervertebral discs consisted of anulus fibrosus and nucleus pulposus.

The transpedicular screw-rod system (screw: diameter 5.5mm, length 40.0mm; rod: diameter 6.0mm, length 51.0mm) and interbody fusion cage (dimension: 22.0mm×10.0mm×9.0mm) models were established by Pro/ENGINEER 2.0 (Parametric Technology Corporation, USA). Then the data were imported into Mimics.

Pars defects were simulated by creating gaps with width of 1mm[17–19]. Both the fractured ends could transmit stresses when they contacted each other during motion. The screw-rod system, interbody fusion cage and spine were assembled and Boolean operated by simulation in Mimics.

The spine was imported into ANSYS 10.0 (ANSYS, Inc. USA) for mesh generation. Then, it was imported back into Mimics to define the material properties referring to previously published literatures. The screw-rod system and cage were directly imported into ANSYS to mesh and define property[20–24]. (Table 1)

**Table 1:** Material properties/values of the FEA models.

	Young’s Modulus (MPa)	Poisson's ratio (μ)
**Vertebral centrum**		
Cortical bone	12000.0	0.3
Cancellous bone	100.0	0.2
End plate	12000.0	0.3
**Vertebral arch**	3500.0	0.25
**Intervertebral disc**		
Nucleus pulposus	1.0	0.49
Annulus fiber	4.2	0.45
**Implants**		
Screw-rod	110000.0	0.3
Cage	110000.0	0.3

**Fig 1:**
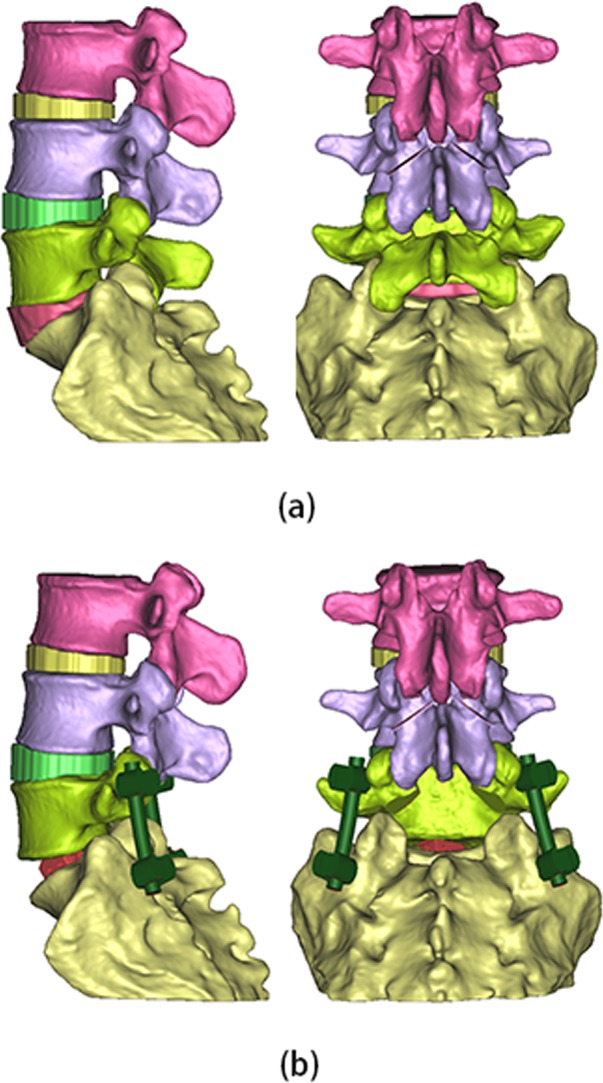
Three-dimensional finite element models. (a) Before surgery model; (b) Short-segment PLIF model.

Two 3D finite element models were established through above process (Fig 1). Before surgery model consisted of 353587 total elements and 581704 total nodal points; Short-segment PLIF model consisted of 536318 total elements and 872853 total nodal points.

### Boundary and Loading Condition

All interfaces in the FEA models were assumed to be bonded except the facet joints which were applied with a surface-to-surface condition of which the friction coefficient was set as 0.1[22, 25]. The nodes of the inferior surface of S1 were completely fixed in all directions[26]. Loading force of 500N[26, 27] was applied on superior surface of L3. Torque of 10N·m was applied to simulate physiological activity in 5 directions: neutral, flexion, extension, lateral bending and axial rotation.

## Results

### Stress on L4 Pars

As indicated in Table 2, the stress on L4 pars was smallest in neutral position, and rose a little in flexion, extension and lateral bending position. The L4 pars suffered the highest stress when the spine rotated. There was no significant difference observed after PLIF surgery was performed at L5-S1 level.

**Table 2:** Comparison of stress on L4 pars, adjacent intradiscal pressure (MPa).

	Stress on L4 pars		L3-L4 intradiscal pressure		L4-L5 intradiscal pressure	
	Before surgery	Short-segment PLIF	Before surgery	Short-segment PLIF	Before surgery	Short-segment PLIF
**Neutral**	73.097	72.034	1.024	1.024	0.640	0.597
**Flexion**	91.366	93.436	1.941	1.940	1.160	1.150
**Extension**	98.177	99.669	0.110	0.109	0.877	0.853
**Lateral bending**	93.590	94.002	0.903	0.904	1.993	2.160
**Axial rotation**	162.075	158.826	0.767	0.786	1.639	1.307

### Adjacent Intradiscal Pressure

The intradiscal pressure of L3-L4 and L4-L5 discs were listed in Table 2. The L3-L4 intradiscal pressure significantly increased when spine flexed forward, and decreased a lot when the spine extended. This change tendency was in accordance with the load distribution shift of spine in vivo, which demonstrated the validation of our model. The change pattern of L4-L5 intradiscal pressure was different from that of L3-L4 disc. The highest pressure presented in lateral bending, and the pressure in flexion and extension were close to each other. After PLIF surgery at L5-S1 level, the L3-L4 intradiscal pressure is very close to that of before surgery. The L4-L5 intradiscal pressure didn’t change much after surgery either, except increased a little in lateral bending (from 1.993 to 2.160 MPa) and decreased a little in axial rotation (from 1.639 to 1.307 MPa). The result indicated that the different pressure change pattern between L3-L4 and L4-L5 intradiscal pressure might due to L4 pars defect rather than the inferior level PLIF surgery.

### Range of Motion

**Fig 2:**
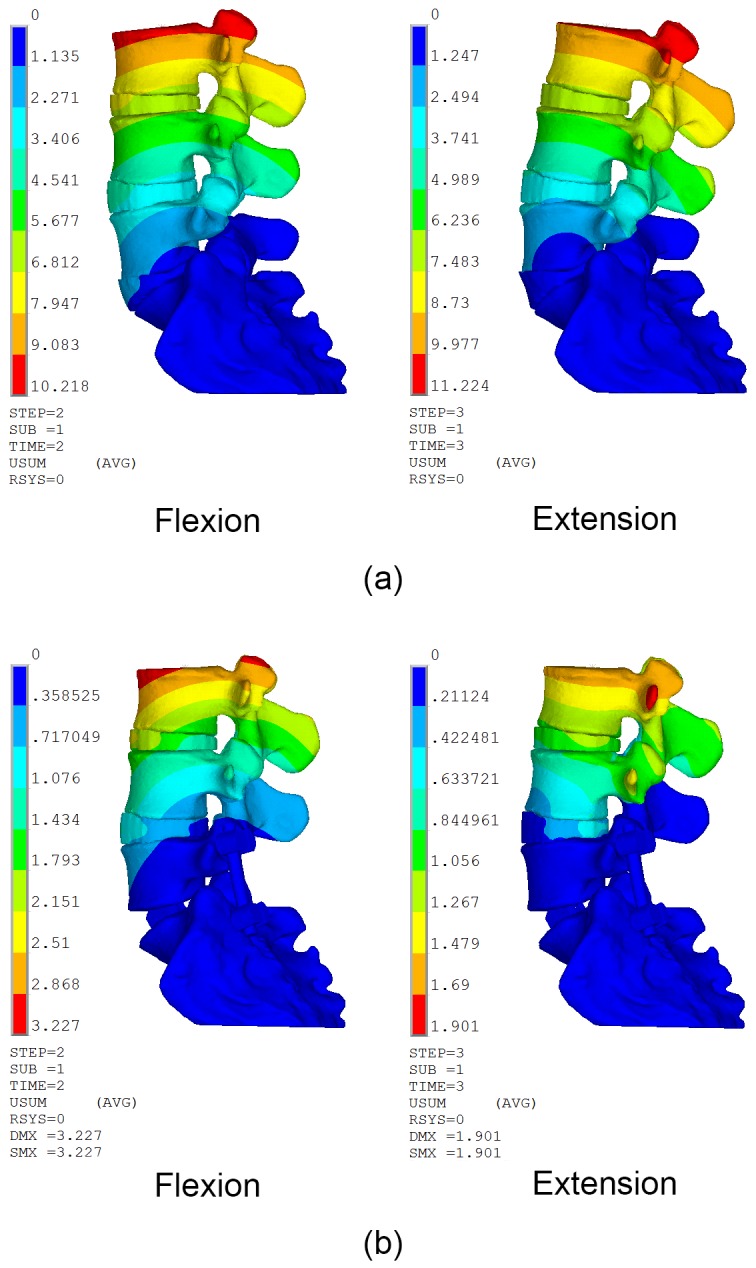
Comparison of displacement distribution in flexion and extension position. (a) Before surgery model; (b) Short-segment PLIF model.

The segmental angles and range of motion (ROM) of L3-L4, L4-L5 and L5-S1 were listed in Table 3 and illustrated in Fig 2. The segmental angle is defined as the intersection angle of the superior end plate of two adjacent vertebras in sagittal plane. The ROM equals to the difference value of segmental angles between flexion and extension position. The segmental angel and ROM had a gradual increase trend from cranial to caudal level. After PLIF surgery, the ROM of L5-S1 disappeared, and that of L3-L4 and L4-L5 both increased only 1 degree.

**Table 3:** Comparison of segmental angels and ROM of L3-L4, L4-L5 and L5-S1 (degree).

	L3-L4		L4-L5		L5-S1	
	Before surgery	Short-segment PLIF	Before surgery	Short-segment PLIF	Before surgery	Short-segment PLIF
**Flexion**	7	7	11	11	24	26
**Extension**	7	8	14	15	32	26
**ROM**	0	1	3	4	8	0

## Discussion

The stability system of vertebral column consists of vertebras, intervertebral discs, ligaments and paravertebral muscles. In spondylolysis, the pars are fractured and detached, so there will almost be no force transferred when the two fractured ends have no contact[28]. The stability of the defected segment will be mainly maintained by the intervertebral disc, ligaments and paravertebral muscles instead. As a chronic disease, adaptive changes might be developed to maintain its stability when the pars fractures. Beutler et al.[14] followed up 30 people diagnosed of pars defect by imageological examination for 45 years. Twelve of them never experienced slippage throughout the follow up period. For those who developed to spondylolisthesis, the average slippage degree is less than 25% (Meyerding grade I), and only three of them underwent lumbar spine surgery. At the final follow-up, the function and pain evaluation of these people had no significant difference with general population of matched age. It indicates that not all of pars defect will develop to spondylolisthesis, and only a few of them will cause severe symptoms that need surgery. Beutler’s report indicates that healthy soft tissues (intervertebral disc, ligaments and paravertebral muscles) are commonly enough to stabilize the lumbar spine. So if the fixation and fusion of the displaced segment will significantly increase the stress on defected pars, intradiscal pressure and ROM of the adjacent segment which has pars defect but no displacement, then a preventative fixation of the adjacent segment for protection is needed. Otherwise, a short-segment fixation and fusion of the displaced segment is enough.

The results of our study indicated that the biomechanical changes at L4 segment were little after a PLIF surgery was performed at L5-S1 level in bi-level spondylolysis. First, the stress on defected L4 pars didn’t have a significant change in all directions of motion after surgery. So it means the surgery will not apply more stress on L4 pars. Second, the intradiscal pressure of discs either superior or inferior to the L4 vertebra didn’t significantly increase after surgery. So it will not lead to higher risk of adjacent disc degeneration. Third, the changes of the ROM of L3-L4 and L4-L5 were very tiny after surgery. So it would not impose a heavier burden on the intervertebral ligaments and paravertebral muscles to maintain stability. All these comparisons demonstrated that the fixation and fusion of L5-S1 level would not increase the risk for the defected L4 segment to develop to spondylolisthesis.

Interestingly, the L4-L5 intradiscal pressure and ROM didn’t increase a lot after a PLIF surgery was performed at L5-S1 level. This does not agree with previous studies which stated that the interbody fusion will increase the adjacent level intradiscal pressure and ROM[29–31]. We think it is because that previous studies were based on intact spine, while our study is the first to investigate the influence of PLIF on an adjacent segment with pars defect. As discussed above, the rigid bony connection will be lost when the pars fractures, the alternative soft tissue connection is resilient. Maybe this resilient connection mechanism can avoid stress concentration at the adjacent level. This hypothesis is partly supported by both experiment and clinical trials. It has been observed that if the adjacent segment above a rigid instrumented level was fixed with a semi-constrained instrument, the rise of ROM and intradiscal pressure at this adjacent segment could be reduced[32, 33]. A similar clinical approach which is called “Topping-off technique” has been demonstrated to restrict the hyper-extension movement of adjacent segments, prevent back and forth movement of proximal vertebrae, and decrease loads of intervertebral disc and facet joints[34]. However, the pars of the non-rigid fixed adjacent segment were intact in these studies, so the validation of our hypothesis needs to be further investigated.

According to the results of our study, if adjacent disc has no apparent degeneration sign in preoperative examinations, a short-segment PLIF should be recommended for the multi-level spondylolysis patients. Short-segment fusion has many advantages compared with long-segment fusion. It can preserve more ROM, has less chance of adjacent level degeneration, less surgery time and blood loss, less cost, et al[35]. Some surgeons chose direct repair instead of interbody fusion in order to preserve the segmental mobility[6], but the bony union rate of direct repair is not satisfying[36, 37]. The most negative influence of long-segment surgery is that it will cause unnecessary iatrogenic injury of ligaments and paravertebral muscles, which are very important stabilizing structures especially in spondylolysis[38, 39]. Additionally, postoperative rehabilitation exercises should be emphasized, so as to enhance the strength of paravertebral muscles.

Our study justified the short-segment fixation for multi-level spondylolysis patients for the first time. In previous reports, all surgeons chose to fix all the defected segments including those without displacement. We think they concerned more about risk aversion while making this choice. Surgeons will prone to overtreatment in case of operative complications. Our study provides biomechanical proof to support short-segment PLIF which can maximize the benefit of these patient. It will be helpful for surgeons to choose proper surgical method for these patients in the future.

There are a few things that should be noted. First, this is purely a FEA study. Although this method is wildly accepted for studying the biomechanical effects in vivo, the parameters or boundary conditions may not perfectly mimic the real properties, which may cause biases. We will try to use biomechanical test on cadavers to verify our FEA results later on. Second, we didn't include a long-segment PLIF model, because the purpose of our study is not to determine whether long and short-segment PLIF is better than the other. Third, segmental slippage was not simulated in our study. Because the slippage severity is highly individualized, the influence of slippage is very complicated for comparison and is beyond the content of this study.

## Conclusion

In this study, we established a FEA model with bi-level spondylolysis at L4 and L5 segments, then simulated a PLIF surgery at L5-S1 level. We performed three dimensional finite element analysis to study the biomechanical changes before and after surgery on this model. Neutral, flexion, extension, lateral bending and rotation position were simulated in both models. The stress on L4 pars, L3-L4 and L4-L5 intradiscal pressure, ROM of L3-4, L4-L5 and L5-S1 levels were evaluated in each position. The results showed that the short-segment PLIF at L5-S1 level would not cause significant biomechanical changes at adjacent L4 segment with pars defect in the bi-level spondylolysis FEA model. So a preventative long-segment fixation is not necessary in multi-level spondylolysis as long as there are no soft tissue degeneration signs at adjacent levels.
